# GeneAnalytics Pathways and Profiling of Shared Autism and Cancer Genes

**DOI:** 10.3390/ijms20051166

**Published:** 2019-03-07

**Authors:** Alexander P. Gabrielli, Ann M. Manzardo, Merlin G. Butler

**Affiliations:** Departments of Psychiatry, Behavioral Sciences & Pediatrics, University of Kansas Medical Center, Kansas City, KS 66160, USA; a228g039@kumc.edu (A.P.G.); amanzardo@kumc.edu (A.M.M.)

**Keywords:** autism spectrum disorders (ASD), cancer, overlapping genes and gene profiling, super-pathways, phenotypes and diseases, molecular functions and processes

## Abstract

Recent research revealed that autism spectrum disorders (ASD) and cancer may share common genetic architecture, with evidence first reported with the *PTEN* gene. There are approximately 800 autism genes and 3500 genes associated with cancer. The VarElect phenotype program was chosen to identify genes jointly associated with both conditions based on genomic information stored in GeneCards. In total, 138 overlapping genes were then profiled with GeneAnalytics, an analysis pathway enrichment tool utilizing existing gene datasets to identify shared pathways, mechanisms, and phenotypes. Profiling the shared gene data identified seven significantly associated diseases of 2310 matched disease entities with factors implicated in shared pathology of ASD and cancer. These included 371 super-pathways of 455 matched entities reflecting major cell-signaling pathways and metabolic disturbances (e.g., CREB, AKT, GPCR); 153 gene ontology (GO) biological processes of 226 matched processes; 41 GO molecular functions of 78 matched functions; and 145 phenotypes of 232 matched phenotypes. The entries were scored and ranked using a matching algorithm that takes into consideration genomic expression, sequencing, and microarray datasets with cell or tissue specificity. Shared mechanisms may lead to the identification of a common pathology and a better understanding of causation with potential treatment options to lessen the severity of ASD-related symptoms in those affected.

## 1. Introduction

Autism spectrum disorders (ASD) include an array of conditions arising from neurodevelopmental defects during a crucial stage of brain formation characterized by deficits in communication ability, a paucity of social skills, repetitive behaviors, and narrow interests [1]. Environmental factors and perinatal care may play a role in both ASD onset and severity, but often arises from a substantial genetic burden. Colvert et al. [2] found heritability estimates of 56% to 95% among monozygotic twins, with additive genetic factors comprising a significant share of the burden. 

Morphologically, the brains of individuals diagnosed with ASD contain abnormal neuronal growth patterns, including an overabundance of neurons and unusual dendritic spine profiles [3,4]. Butler and others in 2005 [5] suggested that common causal factors could contribute to both abnormal neuronal development, autism, and risk for malignancy in patients with autism, with macrocephaly and *PTEN* gene mutations seen in about one-fifth of affected individuals. *PTEN* is an important tumor-suppressor gene reported to play a role in tumor growth, hamartoma disorders (e.g., Cowden, Proteus, Bannayan–Riley–Ruvalcaba syndrome), overgrowth, and cancer [6,7,8]. Several of these overgrowth-related disorders are also at risk of developing malignancy, particularly colorectal cancer.

The genetic architecture of ASD was extensively surveyed through genome-wide association studies (GWAS) and candidate gene approaches [9,10,11]. Several pathways and mechanisms were proposed as mediating factors in the pathogenesis of disorders, although the causative agents of the vast majority of cases remain elusive. However, cross-talk between the canonical Wnt pathway, the Notch signaling cascade [12], and other disturbed genes and pathways [13,14] are believed to play a potential role and helpful in explaining an association with malignancy [15,16]. Furthermore, MAPK and calcium signaling pathways, particularly overlapping calcium-PKC–Ras–Raf–MAPK/ERK processes are strongly associated with ASD. These pathways play a central role in a large range of biological processes and, when abnormal, may compromise biological output and contribute to neuropsychiatric disorders, cell growth, and malignancy [17,18,19,20].

The *PTEN* gene product behaves as an inhibitor of the phosphoinositol 3-kinase/AKT pathway [21]. Disturbances contribute to dysregulation in the pathway leading to excessive uncontrolled cell growth and malignancy. Butler et al. [5] and Varga et al. [21] previously reported elevated frequencies of heterozygous germline *PTEN* gene mutations in the ASD population, suggesting that mutations serve as a critical component of the shared cancer and ASD etiology requiring further research. Investigation into the connection of other genetic factors contributing to ASD and cancer could be of clinical utility. Individuals diagnosed with autism should be screened more frequently for cancers for which they may have a genetic susceptibility. 

In the investigation herein, we utilized GeneAnalytics, which incorporates a computer-based bioinformatics pipeline developed by GeneCards, to identify and interrogate shared genes and their architecture between cancer and ASD. We explored influential shared pathways in overlapping genes which could represent potential therapeutic targets. We reviewed reports on the overlap between approximately 800 autism and over 3500 cancer-related genes utilizing this bioinformatics/pathway analysis program. GeneCards was used as an integrated, human genomic database to leverage information from 125 scholarly databases including genetic, transcriptomic, proteomic, functional, and clinical information to interrogate genomic-related data for our study [22,23]. This novel study may supply information helpful to stimulate or address a potential medical or genetic conundrum and may provide a research-based foundation of common pathology in ASD and cancer.

## 2. Results

Using the VarElect program, autism and cancer genes were screened for overlapping units, according to phenotype category. VarElect identified 138 genes in common between the 792 reported known, susceptible, or clinically relevant genes for autism or ASD (17.4%), and 3.9% of approximately 3500 genes implicated in cancer. The 138 genes and their underlying biological functions were profiled by the commercially available GeneAnalytics program, previously validated in studies of random genes to test the interpretation power or relationship to the gene set under investigation [22]. 

**Figure 1:**
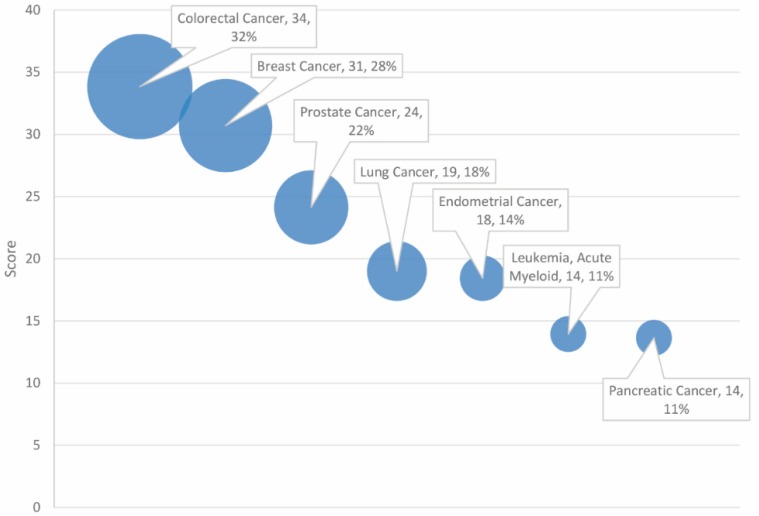
Diseases ranked by score and match rate (size of circle).

The GeneAnalytics output furnished a list of diseases highly associated with the combined cancer and autism gene set. The resulting high score matches (*p* < 0.05) are presented, ranked by score, and categorized by disease type in Table 1. The number of matched genes between the indicated disease and the autism and cancer gene set is included for context. Seven diseases were found to be significantly associated with the dataset, all of which pertain to cancer (see Figure 1). Three of the cancers are reproductive in nature (breast, prostate, and endometrial), while two are gastrointestinal (colorectal and pancreatic). 

**Figure 2:**
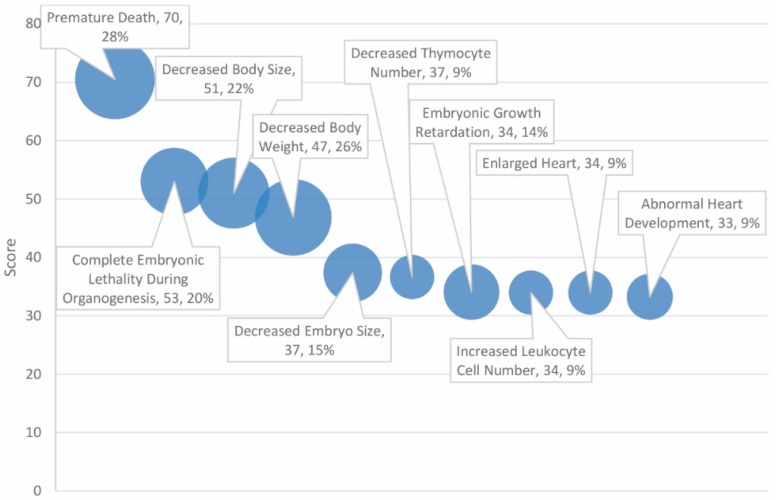
Phenotypes ranked by score and match rate (size of circle) for top ten phenotypes.

Super-pathways were likewise ranked and scored based on degree of association with the gene set. Top-scoring super-pathways are presented with matching rates in Table 2, restricted to the top 30 of 371 significant entries (*p* < 0.05). These included ubiquitous signaling super-pathways such as GPCR (score = 166, 54% match with gene set) and ERK (score = 146, 38% match). Numerous additional signaling super-pathways were found to be significantly associated with the combined gene set, including AKT, HGF development, CREB, cAMP-dependent PKA, fMLP, P70S6K, RET, TGF-β, PEDF induced, MTOR, and PI3K/AKT. Cancer-specific super-pathways, including “glioma” and “pathways in cancer”, were also implicated at a statistically significant level (*p* < 0.05) (see Figure 2).

**Figure 3:**
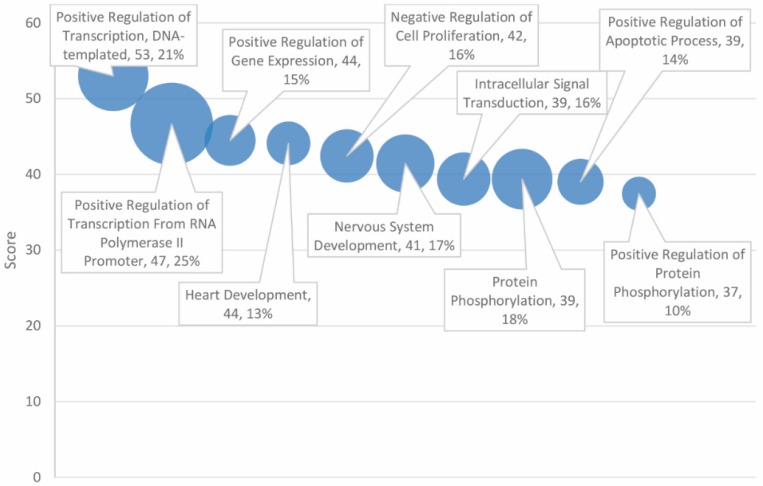
Gene Ontology (GO) biological processes ranked by score and match rate (size of circle) for top ten processes.

Statistically significant Gene Ontology (GO) biological processes are presented in Table 3, which are limited to the top 30 of 153 total high-scoring matches. The top three entries directly relate to the regulation of gene expression. These include “positive regulation of transcription, DNA-templated”, “positive regulation of transcription from RNA polymerase II promoter”, and “positive regulation of gene expression”, which exhibited matching rates of 21%, 25%, and 15%, respectively, with the combined 138 overlapping gene set (see Figure 3). 

**Figure 4:**
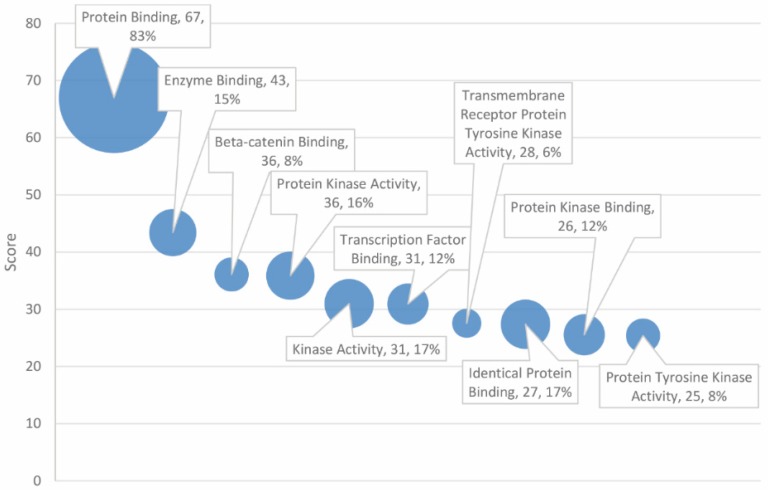
GO molecular function ranked by score and match rate (size of circle) for top ten functions.

The top 30 significant GO molecular functions, of 41 total significant entries, are delineated in Table 4. Prominent entries include protein (score = 67, 83% match rate), enzyme (score = 43, 15% match rate), and β-catenin binding (score = 36, 8% match rate). Kinases and phosphatases were well represented in the analysis as well, with several obtaining high scores, including “protein kinase activity”, “kinase activity”, “TRK activity”, “protein kinase binding”, “protein tyrosine kinase activity”, and “protein phosphatase binding” (see Figure 4).

**Figure 5:**
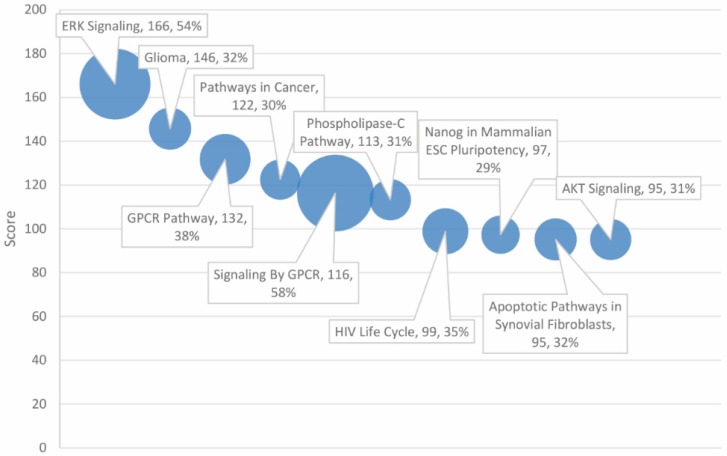
Pathways ranked by score and match rate (size of circle) for top ten pathways.

The 30 top scoring phenotypes, of 145 significant matches, are outlined in Table 5. Phenotypes reflect the importance of ASD and cancer-associated genes in growth and development-related processes (see Figure 5). The top phenotypes include “premature death” (score = 70, 28% match rate), “complete embryonic lethality during organogenesis” (score = 53, 20% match rate), and “decreased body size” (score = 51, 22% match rate). 

## 3. Discussion

As anticipated in our examination of gene data and profiling, the diseases found most in common with the combined autism and malignancy gene set did pertain to cancer. Moreover, colorectal cancer accounted for 32% of overlapping genes, followed by breast and prostate cancer (see Table 1). Two notable exceptions of our own cut-off criteria that may deserve further inspection include Alzheimer disease and amyotrophic lateral sclerosis (ALS), both of which approached but did not attain significance in their association with the gene set, achieving medium scores. Alzheimer disease and ALS are progressive neurodegenerative diseases [23,24,25]. Alzheimer disease and ALS exhibited 11% and 8% overlap with the ASD and cancer gene set, which is a small, though not trivial connection. Enrichment of genes associated with neurodegenerative illnesses among the autism and cancer gene set may be indicative of shared disturbances in neurological growth and development which could contribute to the divergent morphology of autism, cancer, Alzheimer disease, and ALS.

Regarding diseases cited in Table 1, one unusual observation was the lack of representation of brain and neurological diseases. Of the seven high-score matches and six selected borderline (medium-score) matches, only medulloblastoma qualified as a cancer of the brain and nervous system. Reproductive cancers (breast, prostate, and endometrial) and gastrointestinal cancers (colorectal, pancreatic, and hepatocellular carcinoma) were more prevalent. Notably, most of the cancers listed in Table 1 tend to be acquired diseases with a genetic diathesis (e.g., breast and prostate), as opposed to congenital diseases that arise early in development (medulloblastoma). It should also be noted that tumors of the ectoderm and endoderm were disproportionately represented relative to the mesoderm. This may reflect the tissue-specific nature of neural development in the onset of autism. Of note, ASD did not achieve statistical significance with the shared dataset, despite being a requisite for selection and receiving a relatively high-scoring entry. This may be a testament to the complexity of autism spectrum disorder. Even though the gene set was filtered by ASD connection, collectively, the combined genes were more diagnostic of cancer than ASD.

Overall, the highest-scoring super-pathways correlated more strongly with the autism and cancer combined gene set (as evidenced by the higher scores), compared to GO biological processes and GO molecular functions, which were not as strongly correlated, as reflected by their lower scores. The majority of super-pathways (see Table 2) in the top 30 reported entries in the high-scoring category achieved a match rate of at least 20% with the gene set, indicating the relative importance of these super-pathways in the autism and cancer connection. The GeneAnalytics output of the highest scores strongly implicated the ERK signaling pathway in shared pathogenesis based on super-pathway analysis, with a score of 166 and a match rate of 54%, followed by the GPCR pathway at 38%. The MAPK/ERK signaling pathway occupies a key role in mitogen signaling and cell growth with survival [26]. Disturbances in the ERK pathway can disrupt the cell cycle, leading to anomalous cell proliferation and malignant growth. Gene mutations or aberrant functioning in the pathway during early development could contribute to abnormal neuronal growth in ASD. Alterations in GPCR signaling could further contribute to the onset of ASD through aberrant cell signaling and neurotransmitter dysregulation, both of which might lead to changes in cognitive functioning characteristic of ASD. 

For GO biological processes, the three highest-scoring entries pertain to the regulation of gene expression. The outcome of the analysis suggests that an important mechanism in the mutual pathology of autism and cancer may be attributed to variation in transcription factors and nongenic sequences and, hence, differences in the regulation and rate of gene expression. Processes involved in the growth, development, and death of cells were found to be highly associated with the gene set. Indeed, “positive regulation of apoptotic process”, “apoptotic process”, and “negative regulation of apoptotic process” were each flagged as biological processes significantly associated with the combined gene set, with scores of 39 (matching rate of 14%), 37 (18%), and 34 (15%), respectively. Additional growth and death biological processes were also found to be enriched in the gene set, including “heart development”, “negative regulation of cell proliferation”, “nervous system development”, “positive regulation of cell proliferation”, and the “canonical Wnt signaling pathway”.

In terms of the GO molecular functions underlying cancer and autism pathology, the results strongly implicate “protein binding”, which exhibited a score of 67, over 20 points larger than the second, less inclusive entry, “enzyme binding”. Furthermore, 83% of the autism and cancer gene set with 115 out of 138 matched genes was associated with protein binding, suggesting that dysfunction in protein binding may play a significant role in the shared autism and cancer diathesis. In many respects, this paradigm is reflective of the pivotal role of protein binding in the diverse metabolic processes underlying growth and survival. There was a substantial disparity between the high matching rate of protein binding (83%) and that of enzyme binding specifically (15%). The remaining 68% of protein binding that is not explained by enzyme binding and activation by kinase pathways may be attributed to structural protein and signal–receptor binding. This may suggest that structural and signaling mechanisms are disproportionately involved in the shared pathology of cancer and autism as noted previously. MAPK and calcium signaling pathways in genes associated with autism are found in other disorders including cancer [19]. Enzymes may play more of a peripheral role. Enzyme binding overlaps with kinase pathways, many of which are involved in enzyme activation. Kinase and phosphatase activities were also prevalent among the high-scoring entries. Ten of the 30 highest-scoring molecular functions pertain to kinase or phosphatase activity. Both protein binding and kinase activities align with “protein kinase binding”.

The report also implicated β-catenin binding (score of 36; 8% matching rate); β-catenin functions as both a moderator of gene transcription and as an agent of intercellular adhesion [27]. Overexpression of β-catenin, which is encoded by the *CTNNB1* gene, is well characterized as a candidate in the pathogenesis of numerous cancers, as well as heart disease (this may serve as a potential link to the high-scoring GO biological process of “heart development”) (score = 44; match rate = 13%) [28]. The relationship between β-catenin signaling and autism was reported, as β-catenin is dependent on the absence of Wnt [29,30]. Its role in mediating cell adhesion and cellular development suggests it may be a candidate in the pathogenesis of both autism and cancer.

The two highest-scoring phenotypes, “premature death” and “complete embryonic lethality during organogenesis”, reflect the delicacy of neurogenesis and embryonic development. It is likely that only certain combinations of mutations inherited from the autism and cancer gene set can be tolerated and that, at a certain genetic burden, dysfunction in pathways associated with these genes results in irretrievable failure to develop. Designated decreased body size and body weight may also reflect the vital importance of these genes in mediating development, early cell division and number, and the potential consequences of dysfunction.

Three functional pathways are potentially involved, which include genes and pathways for chromatin remodeling, (e.g., *CHD7*, *MECP2*, *DNMT3A*, and *PHF2*), Wnt (e.g., *CHD8*, *PAX5*, and *ATRX*), and other signaling super-pathways (e.g., GPCR, ERK, RET, and AKT) and mitochondrial dysfunction in ASD (e.g., Reference [30]). Theoretically, drugs could be targeted to treat ASD. By developing a rank-ordered list of functional categories significantly associated with autism and cancer using GeneAnalytics pathways and profiling of shared autism and cancer genes, we identified potentially high-impact pathways and common mechanisms which may serve as therapeutic targets in future studies.

The interconnected relationship between autism and cancer invites further investigation in the pathogenesis of the two seemingly unrelated disorders, and it warrants a pharmacological basis of treatment. *PTEN* is an example of a shared gene for autism and cancer and the direct and indirect subject of several formally approved cancer therapeutics including cisplatin, erlotinib, everolimus, cetuximab, and estradiol. For example, cisplatin circumscribes DNA synthesis, activates caspase-3, and facilitates apoptosis, thus rescuing the function of mutant *PTEN* [31]. Considering the role of cisplatin in complementing *PTEN* function, strategic application of the drug could potentially address the autism-associated symptoms arising from *PTEN* dysfunction. Another drug example is everolimus, which behaves as an inhibitor of the mammalian target of rapamycin, and it could impact cancer predisposition and possibly autism development in a manner similar to cisplatin [32].

Although invasive drugs and treatments exist for many types of cancer, a drug is yet to be successfully developed to prevent the onset or progression of ASD symptomology. The unexpected shared etiology with overlapping genes between cancer and autism, particularly those involved in cell-signaling pathways such as MAPK and calcium signaling [19], encourages cautious speculation that, as our knowledge of the staging and mechanisms of ASD improves and applied to animal model testing, the leverage of anti-cancer drugs at minimal dosages could be postulated. For example, the 16p11.2 chromosome deletion is one of the most common copy number variants linked to autism in humans and reportedly involves the *ERK1* gene and other related genes converging in the ERK/MAP kinase pathway. This pathway was the most recognized super-pathway found in our profiling analysis of autism and cancer genes (see Table 2). Perturbations of this pathway can contribute to neuropsychiatric disorders, cell growth, and malignancy; therefore, treatment could lessen these manifestations. To investigate whether pharmacological approaches could be helpful, researchers used a 16p11.2 equivalent deletion mouse model with the *ERK1* gene deleted, and they found that pharmacological inhibitions of ERK signaling (i.e., RB1 and RB3 peptides) rescued cortical cytoarchitecture abnormalities and the abnormal behavioral phenotype associated with this deletion, providing evidence for a potential targeted therapeutic intervention for autism [33]. Hypothetically, treatment could lessen the effects of autism when recognized early and during brain plasticity. 

Furthermore, genetic analysis linked autism with mutations in tumor suppression genes and other cancer-associated genes and pathways reflected in our gene profiling analysis. Important candidate genes (e.g., *PTEN*, *NF1*, and *BRCA2*) are found meeting criteria clearly associated with predisposition to cancer such as colorectal, other gastrointestinal tumors, and breast reported in the top seven autism and cancer-associated diseases (see Table 1). For example, mutations in the *PTEN* gene are linked to colorectal, thyroid, head, neck, prostate, skin, breast, and lung cancer and about 10% of children with autism have *PTEN* gene mutations, making this an important gene to target regarding therapeutic intervention for autism with predisposition to cancer. ASDs and cancer overlap extensively in signal transduction pathways as illustrated by our study, involving metabolic processes; these areas should be targeted by treatment strategies. Such insights may enable more accurate gene-informed cancer risk assessment for targeted therapeutic and medical management.

To date, there were a few preclinical models tested with anticancer medication on ASD symptoms. For example, Kilincaslan et al. [34] reported the beneficial effects of everolimus on autism and attention-deficit hyperactivity disorder (ADHD) symptoms in a group of patients with tuberous sclerosis complex (TSC). This drug inhibits mTOR, one of the top 30 super-pathways identified with high scores when comparing shared autism and cancer genes. It is a treatment for TSC in which autism is a finding, along with renal angiomyolipomas and astrocytomas. The drug reduced tumor growth, decreased seizures, and improved autistic, ADHD, and depression symptoms. However, there is a paucity of studies on the effects of this drug on neuro-psychiatric symptoms, which merit further consideration. Treatment would be a new avenue to pursue and further explore, particularly in those with ASD, the involvement of shared autism and cancer-related genes.

## 4. Materials and Methods 

Shared genetic architecture between cancer and ASD was examined firstly using VarElect, a sequence phenotyper affiliated with GeneAnalytics (Alameda, CA, USA) [35,36]. A reported list of 792 clinically relevant, susceptible, or known genes for ASD, summarized previously by Butler et al. [37], and over 3500 recognized cancer genes found in the GeneAnalytics databases were entered into the VarElect phenotyper program to identify overlapping genes.

The ASD genes were filtered by their association with the query “cancer” and limited to those with established pathways. Based on the above parameters, VarElect produced 138 genes with known connections to both cancer and ASD. The refined list of 138 genes was then entered into GeneAnalytics, a gene set analytical tool which uses GeneCards, an integrated genomic database, to parse genes and their relationships, as previously reported [22,38,39,40].

The GeneAnalytics program produces a report containing seven categories: diseases, pathways, tissues and cells, phenotypes, Gene Ontology (GO) molecular functions, GO biological processes, and compounds related to the query of interest (i.e., cancer and ASD gene). Each entry in these functional categories was sorted with the GeneAnalytics proprietary matching and scoring algorithm following protocols published previously in the study of psychiatric, behavior, and obesity-related genes by our research group [22,39,40]. Only those genes with the highest calculated scores were further analyzed for each of the seven GeneAnalytics categories. In addition to scoring fields based on their relevance to the gene set, fields were subdivided into three categories, “high-score matches”, “medium-score matches”, and “low-score matches”. High-score matches exhibited a corrected *p*-value of less than 0.05, while medium-score matches exhibited a corrected *p*-value between 0.05 and 0.1. For the purposes of this investigation, medium- and low-score matches were excluded from the results. The reporting of high-score matches was restricted to the top 30 entries, in instances where more than 30 were found to be significant (*p* < 0.05). Corrections were made based on multiple testing (Bonferroni correction).

## Figures and Tables

**Table 1:** Profiling of high scores in overlapping genes for autism and cancer-associated diseases.

Score	Disease	Disease Categories	Number of Matched Genes (% of 138 Overlapping Genes)
33.84	Colorectal cancer	Gastrointestinal diseases, genetic diseases, rare diseases, cancer diseases	44 (32%)
30.70	Breast cancer	Reproductive diseases, genetic diseases, rare diseases, cancer diseases	39 (28%)
24.13	Prostate cancer	Reproductive diseases, genetic diseases, rare diseases, cancer diseases	31 (22%)
19.01	Lung cancer	Respiratory diseases, genetic diseases, cancer diseases	25 (18%)
18.43	Endometrial cancer	Reproductive diseases, genetic diseases, rare diseases, cancer diseases	19 (14%)
13.94	Leukemia, acute myeloid	Immune diseases, blood diseases, genetic diseases, rare diseases, cancer diseases	15 (11%)
13.64	Pancreatic cancer	Gastrointestinal diseases, endocrine diseases, genetic diseases, rare diseases, cancer diseases	15 (11%)

**Table 2:** Profiling of high scores in overlapping genes for autism and cancer-associated super-pathways.

Score	Super-Pathways	Number of Total Genes	Number of Super-Pathway Matched Genes	% of 138 Overlapping Genes
166.10	Elk-related tyrosine kinase (ERK) signaling	1177	74	54%
145.73	glioma	313	44	32%
131.74	G-protein coupled receptor (GPCR) pathway	708	53	38%
122.48	Pathways in cancer	395	42	30%
116.32	Signaling by GPCR	2601	80	58%
113.29	Phospholipase-C pathway	498	43	31%
98.91	Human immune-deficiency virus (HIV) life cycle	865	48	35%
97.32	NANOG in mammalian embryonic stem cell pluripotency	533	40	29%
95.18	Apoptotic pathways in synovial fibroblasts	725	44	32%
95.13	AKT murine thymoma viral oncogene homolog (AKT) signaling	681	43	31%
94.06	Development HGF signaling	234	30	22%
90.05	CAMP response element-binding protein (CREB) pathway	528	38	28%
81.54	Integrated breast cancer pathway	154	24	17%
81.30	Proteoglycans in cancer	203	26	19%
80.40	Nuclear factor of activated (NFAT) T cells and cardiac hypertrophy	326	30	22%
78.97	Integrin pathway	568	36	26%
78.26	Development of vascular endothelial growth factor (VEGF) signaling via VEGFR2, generic cascades	147	23	17%
77.70	Activation of cyclic adenosine monophosphate (CAMP)-dependent protein kinase A (PKA)	628	37	27%
77.43	Formyl peptide receptor (FMLP) pathway	317	29	21%
77.00	P70S6K signaling	390	31	22%
74.95	Rearranged during transfection (RET) signaling	974	43	31%
72.42	Transforming growth factor (TGF)-beta pathway	652	36	26%
72.03	Glioblastoma multiforme	111	20	14%
71.00	Pigment epithelium-deprived factor (PEDF) induced signaling	721	37	27%
70.30	P21-activated kinase (PAK) pathway	682	36	26%
69.39	Endometrial cancer	122	20	14%
67.08	Mechanistic target of rapamycin (MTOR) signaling pathway (KEGG)	209	23	17%
66.63	PI3K/AKT signaling pathway	342	27	20%
66.33	Developmental biology	1079	42	30%
65.44	Focal adhesion	283	25	18%

**Table 3:** Profiling of high scores in overlapping genes for autism and cancer-associated Gene Ontology (GO) biological processes.

Score	GO Biological Processes	Number of Total Genes	Number of Matched Genes	% of 138 Overlapping Genes
53.00	Positive regulation of transcription, DNA-templated	596	29	21%
46.66	Positive regulation of transcription from RNA polymerase II promoter	1016	34	25%
44.49	Positive regulation of gene expression	346	21	15%
44.10	Heart development	232	18	13%
42.44	Negative regulation of cell proliferation	419	22	16%
41.44	Nervous system development	535	24	17%
39.38	Intracellular signal transduction	467	22	16%
39.37	Protein phosphorylation	629	25	18%
39.02	Positive regulation of apoptotic process	330	19	14%
37.45	Positive regulation of protein phosphorylation	155	14	10%
36.52	Apoptotic process	690	25	18%
36.16	Positive regulation of sequence-specific DNA-binding transcription factor activity	105	12	9%
34.58	Positive regulation of cell proliferation	500	21	15%
34.22	Negative regulation of apoptotic process	507	21	15%
33.45	Phosphorylation	700	24	17%
31.65	Canonical Wnt signaling pathway	80	10	7%
31.62	Cell adhesion	620	22	16%
31.02	Phosphatidylinositol-mediated signaling	112	11	8%
30.96	Signal transduction	2032	40	29%
29.94	Response to drug	323	16	12%
29.83	Thymus development	44	8	6%
29.33	Visual learning	46	8	6%
28.92	Extracellular matrix organization	203	13	9%
28.09	Protein autophosphorylation	173	12	9%
27.55	Negative regulation of transcription from RNA polymerase II promoter	724	22	16%
27.40	Cell-cycle arrest	143	11	8%
27.05	Regulation of phosphatidylinositol 3-kinase signaling	82	9	7%
26.20	Substrate adhesion-dependent cell spreading	39	7	5%
25.00	Negative regulation of cysteine-type endopeptidase activity involved in apoptotic process	68	8	6%
24.79	Peptidyl-tyrosine phosphorylation	171	11	8%

**Table 4:** Profiling of high scores in overlapping genes for autism and cancer-associated GO molecular functions.

Score	GO Molecular Functions	Number of Total Genes	Number of Matched Genes	% of 138 Overlapping Genes
66.97	Protein binding	9013	115	83%
43.40	Enzyme binding	360	21	15%
36.09	β-Catenin binding	80	11	8%
35.87	Protein kinase activity	530	22	16%
30.98	Kinase activity	698	23	17%
30.91	Transcription factor binding	308	16	12%
27.55	Transmembrane receptor protein tyrosine kinase activity	54	8	6%
27.38	Identical protein binding	797	23	17%
25.54	Protein kinase binding	402	16	12%
25.40	Protein tyrosine kinase activity	164	11	8%
23.70	Transcription regulatory region DNA binding	229	12	9%
21.10	Phosphatidylinositol-4,5-bisphosphate 3-kinase activity	66	7	5%
20.68	Protein phosphatase binding	69	7	5%
20.58	RNA polymerase II Core promoter proximal region sequence-specific DNA binding	336	13	9%
20.43	Protein C-terminus binding	185	10	7%
19.66	Ubiquitin protein ligase binding	299	12	9%
18.88	Transcriptional activator activity, RNA polymerase II core promoter proximal region sequence-specific binding	260	11	8%
17.81	Repressing transcription factor binding	34	5	4%
17.69	Protein homodimerization activity	758	18	13%
17.69	Chromatin binding	404	13	9%
17.45	Cell adhesion molecule binding	63	6	4%
17.27	C–X3–C chemokine binding	5	3	2%
16.82	Protein heterodimerization activity	495	14	10%
16.48	Nitric-oxide synthase regulator activity	6	3	2%
16.19	Androgen receptor binding	43	5	4%
15.95	Protein domain specific binding	264	10	7%
15.57	Transferase activity	1759	28	20%
15.54	Receptor binding	398	12	9%
15.50	Nuclear hormone receptor binding	23	4	3%
15.28	Transcription factor activity, sequence-specific DNA binding	1029	20	14%

**Table 5:** Profiling of high scores in overlapping genes for autism and cancer-associated phenotypes.

Score	Phenotypes	Number of Total Genes	Number of Matched Genes	% of 138 Overlapping Genes
70.49	Premature death	832	39	28%
53.00	Complete embryonic lethality during organogenesis	540	28	20%
51.00	Decreased body size	742	31	22%
46.85	Decreased body weight	1144	36	26%
37.37	Decreased embryo size	450	21	15%
36.64	Decreased thymocyte number	102	12	9%
34.08	Embryonic growth retardation	404	19	14%
33.98	Increased leukocyte cell number	120	12	9%
33.98	Enlarged heart	120	12	9%
33.25	Abnormal heart development	158	13	9%
33.05	Decreased B-cell number	196	14	10%
29.78	Partial embryonic lethality during organogenesis	234	14	10%
29.60	Partial postnatal lethality	546	20	14%
29.50	Increased tumor incidence	124	11	8%
28.15	Enlarged spleen	256	14	10%
27.97	Abnormal sensory neuron innervation pattern	52	8	6%
27.76	Abnormal rostral–caudal axis patterning	53	8	6%
27.60	Complete prenatal lethality	264	14	10%
27.18	Partial perinatal lethality	225	13	9%
27.13	Hyperactivity	271	14	10%
27.09	Abnormal blood vessel morphology	146	11	8%
27.04	Increased mammary adenocarcinoma incidence	20	6	4%
26.94	Complete lethality throughout fetal growth and development	186	12	9%
26.90	Abnormal response/metabolism to endogenous compounds	83	9	7%
26.76	Complete postnatal lethality	378	16	12%
25.78	Abnormal B-cell differentiation	91	9	7%
25.38	Abnormal definitive hematopoiesis	94	9	7%
24.21	Abnormal heart morphology	178	11	8%
24.05	Decreased sensory neuron number	14	5	4%
23.89	Partial prenatal lethality	274	13	9%

## Abbreviations

ASD	Autism spectrum disorders
GWAS	Genome-wide association studies
RNA	Ribonucleic acid
DNA	Deoxyribonucleic acid
GO	Gene ontology
KEGG	Kyoto Encyclopedia of Genes and Genomes
NICHD	National Institute of Child Health and Human Development
